# The role of point-of-care viral load monitoring in achieving the target of 90% suppression in HIV-infected patients in Nigeria: study protocol for a randomized controlled trial

**DOI:** 10.1186/s12879-019-3983-6

**Published:** 2019-05-02

**Authors:** Seema T. Meloni, Oche Agbaji, Charlotte A. Chang, Patricia Agaba, Godwin Imade, Stephen Oguche, Ahmed Mukhtar, Kiren Mitruka, Mackenzie Hurlston Cox, Aaron Zee, Phyllis Kanki

**Affiliations:** 1 Department of Immunology & Infectious Diseases, Harvard T. H. Chan School of Public Health, 651 Huntington Avenue, Boston, MA USA; 2 Jos University Teaching Hospital, Bauchi Road, PMB, Jos, Plateau State 2076 Nigeria; 3 Centers for Disease Control and Prevention, Plot 1075 Diplomatic Drive, Abuja, Nigeria; 4 Centers for Disease Control and Prevention, 1600 Clifton Road, Atlanta, GA USA

**Keywords:** HIV, Antiretroviral therapy, Viral load, Point-of-care, Retention, Adherence, Nigeria

## Abstract

**Background:**

The Joint United Nations Programme on HIV/AIDS 90–90-90 goal envisions 90% of all people receiving antiretroviral therapy to be virally suppressed by 2020. Implied in that goal is that viral load be quantified for all patients receiving treatment, which is a challenging undertaking given the complexity and high cost of standard-of-care viral load testing methods. Recently developed point-of-care viral load testing devices offer new promise to improve access to viral load testing by bringing the test closer to the patient and also returning results faster, often same-day. While manufactures have evaluated point-of-care assays using reference panels, empiric data examining the impact of the new technology against standard-of-care monitoring in low- and middle-income settings are lacking. Our goal in this trial is to compare a point-of-care to standard-of-care viral load test on impact on various clinical outcomes as well to assess the acceptability and feasibility of using the assay in a resource-limited setting.

**Methods:**

Using a two-arm randomized control trial design, we will enroll 794 patients from two different HIV treatment sites in Nigeria. Patients will be randomized 1:1 for point-of-care or standard-of-care viral load monitoring (397 patients per arm). Following initiation of treatment, viral load will be monitored at patients’ 6- and 12-month follow-up visits using either point-of-care or standard-of-care testing methods, based on trial assignment. The monitoring schedule will follow national treatment guidelines. The primary outcome measure in this trial is proportion of patients with viral suppression at month 12 post-initiation of treatment. The secondary outcome measures encompass acceptability, feasibility, and virologic impact variables.

**Discussion:**

This clinical trial will provide information on the impact of using point-of-care versus standard-of-care viral load testing on patient clinical outcomes; the study will also supply data on the acceptability and feasibility of point-of-care viral load monitoring in a resource-limited setting. If this method of testing is acceptable and feasible, and also superior to standard of care, the results of the trial and the information gathered will inform future scaled implementation and further optimization of the clinic-laboratory network that is critical for monitoring achievement of the 90–90-90 goals.

**Trial registration:**

US National Institutes of Health Clinical Trials.gov: NCT03533868. Date of Registration: 23 May 2018. Protocol Version: 10. Protocol Date: 30 March 2018.

## Background

The Joint United Nations Programme on HIV/AIDS (UNAIDS) 90–90-90 goals to end the acquired immunodeficiency syndrome (AIDS) epidemic by 2030, where the third target envisions 90% of all people receiving antiretroviral therapy (ART) virally suppressed, represents an ambitious strategy [1]. The World Health Organization (WHO) estimated that if all countries aim to achieve the 90–90-90 targets, the demand for VL tests in 2018 would reach about 27 million [2]. Viral load (VL) quantification is the optimal method of monitoring viral suppression, but its usage is challenging, since it requires a sophisticated laboratory assay and is associated with numerous logistical hurdles, including acquisition of cold-chain dependent kits. The larger VL testing platforms have sufficient capacity to conduct testing; however, the systems are not robust enough to support their efficient use as some specimens must be transported over long distances for testing, an estimated 10% of the machines are not operational, and oftentimes testing locations are faced with shortage of reagents. For treatment sites in remote, resource-limited settings (RLS), and those without access to conventional testing methods, these issues contribute to high turnaround times (TAT), with subsequent delays in patients receiving results and, potentially appropriate care [3].

The development of an affordable, reliable, point-of-care (POC) VL assay has been considered a “game-changer,” where increased access, minimal laboratory worker training, and same day results can be addressed in a single solution. Same day results may encourage adherence, earlier detection of virologic failures (VF), and timelier switch to second-line (2 L) regimens and improved clinical outcomes. The currently developed POC VL assays are in varying stages of evaluation, including manufacturer’s evaluations to verify device performance, independent laboratory evaluations, and in-country performance evaluations. Prior to broader implementation in low- and middle-income countries (LMIC), it is important to evaluate the impact of this new technology against the standard-of-care (SOC) method of VL monitoring in an actual LMIC ART setting.

Nigeria, a country with a population of nearly 180 million, has the second highest burden of human immunodeficiency virus (HIV) in the world, serves as a relevant setting for testing feasibility and efficacy of POC VL monitoring. In 2016, UNAIDS estimated there were 3.2 million people living with HIV (PLHIV) in Nigeria, of which 1.1 million (34%) knew their HIV status and 970,000 (30%) were on ART [4]. As of April 2018, there were 27 VL instruments in Nigeria, despite the fact that the Nigerian National ART Taskforce recommends the use of VL monitoring for patients on ART [5]. Even if all patients on ART could be monitored with VL, it would mean that each laboratory with a VL instrument would need to monitor close to 30,000 patients, with many of those samples traveling long distances. With published estimates of median TAT sample collection to VL result receipt at site ranging from 3 days in Kenya to 50 days in Cote d’Ivoire [6–9], shifting to use of POC VL testing in Nigeria has the potential to reduce overall test TAT [5]. Further optimizing the laboratory-clinic network by adding POC VL technology where needed could potentially increase the proportion of patients that will maintain a suppressed VL.

In order to present the case for implementing POC VL testing as part of the viral load testing strategy in various sites across Nigeria and other LMICs, data on the feasibility and efficacy of using POC testing for VL monitoring are needed. To address this need, we have designed a randomized controlled trial comparing SOC, as described by the Nigerian National Guidelines, to POC VL monitoring methods to provide operational evidence for implementation of POC VL testing in Nigeria. We anticipate the results of this analysis will have further relevance for ART programs in most LMICs.

### Study objectives

This trial seeks to optimize VL monitoring. We will conduct a randomized controlled trial of POC VL monitoring using the Cepheid Xpert HIV-1 Viral Load (Xpert HIV-1 VL) assay, run on the GeneXpert platform, versus SOC VL monitoring in patients newly initiating ART with the goal of providing empiric evidence that use of POC VL monitoring is an effective, acceptable, and feasible approach to optimizing VL monitoring.

The Xpert HIV-1 VL assay is a quantitative assay with a quantification range of 40 to 10,000,000 copies/mL that provides a visual read-out within 90 min. The Xpert HIV-1 VL assay has been validated in a number of performance evaluations of clinical plasma samples in Africa [10–12]. The Xpert HIV-1 VL assay received Conformité Européene-In Vitro Diagnostic marking in December 2014, and received WHO prequalification of in vitro diagnostics in July 2017 [13].

## Methods/design

### Study design

**Fig. 1:**
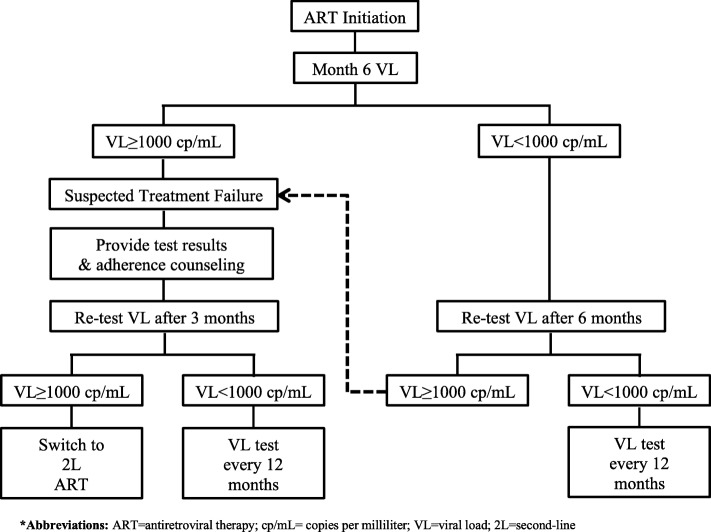
Adaptation of Nigerian National Guideline recommended algorithm for VL testing in ART patients

**Fig. 2:**
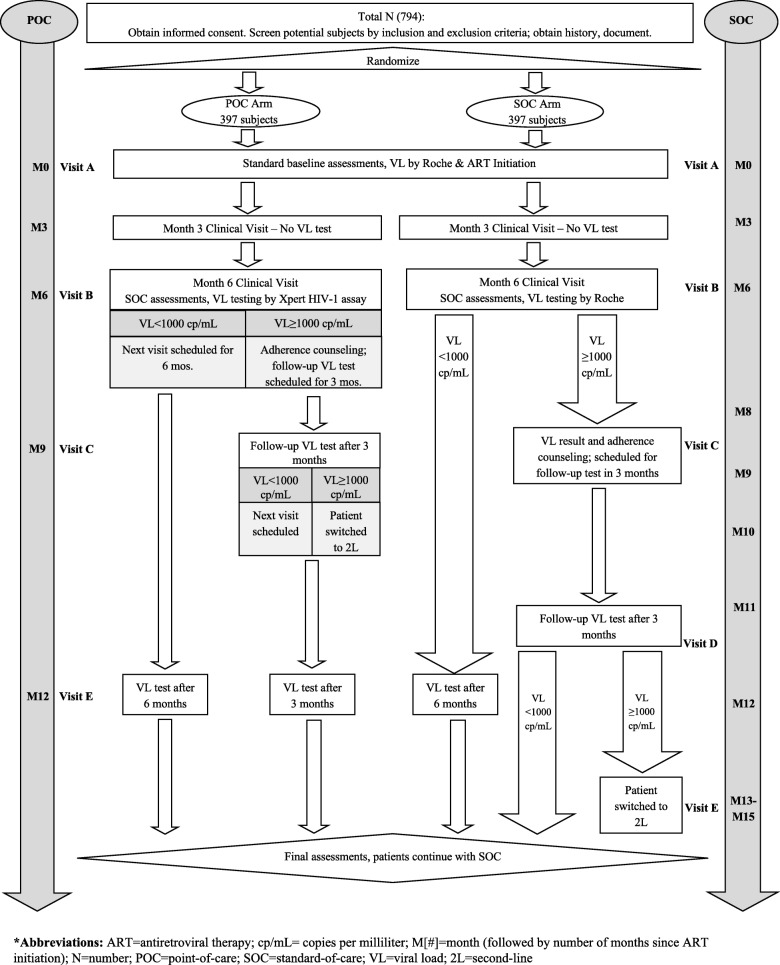
Schematic of Study Design

We will conduct an un-blinded randomized implementation trial comparing POC VL monitoring, using the Xpert HIV-1 VL assay, to SOC VL monitoring, using the Roche AmpliPrep/COBAS TaqM (CAP/CTM) HIV-1 test, v2.0 (Roche VL) system. VL monitoring will occur according to the current Nigerian ART guidelines recommended algorithm (Fig. 1) with the addition of a baseline VL test (Fig. 2). The primary outcome is proportion of patients with virologic suppression (VL < 1000 copies/mL) at 12 months post-initiation of ART; as such, patients will be informed that participation in this trial entails follow-up to the month 12 visit, after which patients will continue to receive the SOC services to which they were entitled as patients of the clinic.

### Study setting

The study will be conducted at two sites in Plateau State, Nigeria: Jos University Teaching Hospital (JUTH), a tertiary teaching hospital with more than a decade of experience in ART and VL monitoring, and their satellite clinic, the Comprehensive Health Centre, Zamko (CHCZ), a secondary district hospital. Both sites are affiliated with APIN (AIDS Prevention Initiative Nigeria) Public Health Initiatives (PHI) program and receive U.S. President’s Emergency Plan for AIDS Relief (PEPFAR) funding. JUTH has been receiving PEPFAR support since 2004, through a partnership established between the Harvard T. H. Chan School of Public Health and APIN PHI. CHCZ started receiving Harvard/APIN PEPFAR funds as a scale-up program of JUTH in 2007.

All SOC VL testing by Roche will be conducted at JUTH, as is currently the standard practice. Samples for SOC VL testing from CHCZ will be transported to JUTH on a regular basis and SOC VL results will be transmitted electronically in a password-protected encrypted data file from the JUTH Data Manager to the CHCZ Data Manager for entry into the patient records.

### Study population

The proposed sample size is 794 patients newly initiating ART (397 per arm). The inclusion criteria for the study is all HIV-infected patients initiating ART. Exclusion criteria are: 1) previous ARV experience; and, 2) women who are pregnant at ART initiation. As of the study initiation, JUTH had an average of 36–48 new enrollees per month and CHCZ about 19 new patients per month; combined, the target sample size of 794 should be reached by 18 months of patient recruitment.

### Recruitment and consent

As part of the ongoing clinical ART program at JUTH and CHCZ, adult and pediatric patients over 18 months of age who test newly positive for HIV by rapid test and pediatric patients under 18 months of age who test positive for HIV by a DNA polymerase chain reaction test can enroll into the HIV care program following informed consent for care. Upon enrollment in the ART program, each patient is assigned a unique coded patient identification (ID) number. For this trial, we will utilize the same ID numbers that are used at the site for general HIV care to ensure that no data are separated from the existing system of record keeping.

During standard ART clinic patient enrollment procedures, the adherence counselor for the trial at each site will verify if the patient is eligible for trial participation (Fig. 2); then, following a standardized recruitment script, the counselor will share information about the trial with the patient and request participation. Each patient will be informed of the randomization process and that he or she might be placed in either the SOC or POC group. They will also be informed that they will not be blinded to the assignment. Patients will also be informed that if they choose to withdraw from the trial at any time, they will retain all services entitled to them in their enrollment in the JUTH/CHCZ ART PEPFAR program. They will continue to receive the SOC services to which they were already entitled as part of their regular services. If the patient (or guardian, in the case of children) consents to participate, the adherence counselor will ask that they complete an informed consent form.

### Randomization

Upon written informed consent, patients initiating ART at each clinic will be randomized on a 1:1 basis, with separate randomization of adult and pediatric patients, to the SOC VL monitoring control arm or the POC VL monitoring arm (Fig. 2). The randomized lists were created prior to the start of the trial using a permuted block randomization process to ensure balance across treatment groups. Block size was based on the average weekly enrollment rates at the respective sites in the 8 weeks preceding the trial start date. The adherence counselor will use these lists and sequentially assign patients to the next allocation listed, making note of assignments that were made.

### Sources of materials

For SOC, plasma is extracted from whole blood drawn by venipuncture for Roche VL determination. At the month 6 and 12 visits, patients in the POC arm will undergo the same venipuncture blood draw with subsequent extraction of plasma from the blood sample. This trial will include an additional venipuncture for Roche VL determination on the date of ART initiation in order to establish baseline VL for all patients regardless of randomization. We will maintain all excess collected samples in a repository along with other relevant clinical data from patients that will be described in the informed consent.

In addition, three surveys will be administered for this trial: a baseline adherence and retention survey for all enrolled patients, a post-trial survey for patients in the POC arm, and a post-trial survey for healthcare workers (HCWs). The post-trial surveys will collect information on acceptability of and satisfaction with the POC assay and monitoring from the perspectives of both patients and providers.

Clinical data collected from trial subjects will be entered onto standardized clinical forms and transferred to electronic databases already in use at the sites and be utilized for trial analyses. Data from all trial surveys will be entered into separate electronic databases that correspond to the individual forms.

### Standard of care procedures

After providing informed consent for care and receiving their patient ID, newly diagnosed HIV-positive patients are pre-assessed and HIV status is confirmed. The patient will undergo a clinical assessment, including: tuberculosis (TB) screening with sputum (with or without chest radiograph); review of other HIV-related diagnoses; collection of medical history and current medications; provision of any relevant referrals and basic laboratory tests (complete blood count with platelets, alanine aminotransferase, creatinine, urea, glucose, cholesterol, hepatitis status, and CD4+ cell count. Following confirmation of TB status, the patient will be initiated on ART. Patients are asked to make monthly ART refill pick-ups at the clinic pharmacy. Once enrolled in the ART program, patients are followed up with clinic visits at 3 and 6 months, or more, as medically indicated. Per SOC, VL is monitored from the samples collected at the month 6 and 12 visits and then every 12 months thereafter if the patient is virologically suppressed (Fig. 1). All clinical data are recorded on standardized forms and entered into an electronic medical record system maintained and managed on-site.

### Description of the intervention

Patients enrolled in the trial will follow the SOC clinical visit protocol, where they make scheduled clinic visits at months 6 and 12 post-initiation of ART; at those visits, samples will be drawn for VL monitoring. For the month 6 and 12 visits, patients that are enrolled in the POC VL monitoring arm will be asked to wait for their VL results for approximately 2–3 h, overlapping with routine clinical waiting times. Participants in the POC arm will receive their Xpert HIV-1 VL test results on the same day as their month 6 and 12 visits. If a patient in the POC arm is unable to wait for the test result on their visit date (e.g., if the Xpert HIV-1 VL assay produces an invalid result and the assay needs to be conducted again), the patient will complete their clinical visit with their physician, obtain their pharmacy prescription, and receive any additional relevant services; once the test result is available, the study coordinator will call them with their result; those patients with unsuppressed VLs will be re-scheduled to return in the next few weeks, as early as the patient can return. All other aspects of trial patient HIV care and treatment will be identical to services that non-trial patients normally receive at these treatment centers.

For patients with suppressed VL, no immediate action will be taken and patients will be expected to return for their next clinical follow-up visit in 6 months and informed to continue making their monthly ART refill pick-ups at the pharmacy. For POC patients, VL results will be communicated same day and for SOC patients, results will be shared with the patient at the next follow-up visit.

Patients whose VL is not suppressed (≥1000 copies/mL) will receive their results from their clinician once available, be counseled about their test result and educated about the importance of adherence, and scheduled to return in 3 months. At the next visit, the patient will be directed to phlebotomy for venipuncture. POC patients will receive their VL results the same day from their clinician; if their second follow-up VL is unsuppressed, indicating the patient is in VF, the clinician will make the decision to switch them to a 2 L ART regimen. For SOC patients, if the follow-up VL test is not suppressed, the patient will be contacted and asked to return to the clinic so that the clinician can make the determination if the patient must be switched to 2 L ART regimen.

### Subject withdrawal or termination

Subjects may withdraw voluntarily from the trial. Participation may also be terminated by the JUTH Principal Investigator (PI) due to adverse clinical event, laboratory abnormality, or other medical condition or situation occurs such that continued participation in the trial would not be in the best interest of the subject. Subjects who withdraw from the trial will retain all services entitled to them in their enrollment in the JUTH/CHCZ ART PEPFAR program. They will continue to receive the SOC services to which they were already entitled as part of their regular services.

### Trial endpoint

In this trial, patients will be followed up to their month 12 visit or up to date of 2 L switch for SOC participants with two consecutive unsuppressed VLs, which may occur up to 15 months after ART initiation (Fig. 2). Following that time point, the patients will continue receiving HIV care and treatment utilizing the SOC procedures for the clinic. At the endpoint, we will ask patients, caregivers of patients under the age of 18 years, and providers for their participation in surveys on the operational performance and acceptability of the POC versus SOC VL monitoring. Acceptability questions for the patients focus on patients’ perception of the extra time they had to wait for test results and how they feel about receiving their results on the same day as their clinical visit. Questions for HCW focus on their thoughts about using the assay, how easy it was to use and interpret the results, and their perception of how their patients liked having same-day results.

### Participant retention

If a patient is late for a follow-up visit, their record will be flagged. A list containing the names and IDs of all patients that are late for their visits will be generated daily by the medical records officer responsible for scheduling the appointments. The list will be provided to the tracking team at the clinic. The tracking team will initially contact the patients through a phone call; if the team reaches the patient, they will encourage the patient to return to the clinic for their visit. However, if the team is unable to track the patient via phone, they will pay a visit to the patient’s registered address to ascertain why the patient had not returned for their clinic visit. Once the patient is located, they will be scheduled for their follow-up visit. Any information about why the patient has not returned will be documented in the patient chart.

### Data collection and laboratory procedures

Standard processing of samples will include proper plasma separation and automated extraction, amplification, detection, and VL quantification in copies/mL or log copies, following manufacturer’s specifications of the respective POC and SOC VL assays.

All specimens will be collected according to routine care protocols as dictated by the Nigerian National Guidelines [14], with an additional Roche VL test at ART initiation for baseline assessment. For VL testing, whole blood is collected by standard venipuncture in blood collection tubes. The sample will be sent to the laboratory along with the order form that indicates that the sample will undergo VL quantitation using either the POC Xpert HIV-1 VL assay or the SOC Roche method. All POC Xpert HIV-1 VL assays will be performed on site, where the sample was collected (JUTH or CHCZ). All Roche assays will be performed at JUTH; SOC samples collected at CHCZ will be transported to JUTH. All samples will be processed for plasma. Extra plasma will be stored in the − 70 **°**C freezer for future use as needed.

For the POC VL test, the Cepheid GeneXpert device analyzes the sample and provides VL results in approximately 90 min. If the test produces an invalid result for the trial patients, a new sample will be run using extra stored plasma.

JUTH has the established infrastructure and experience with drug resistance mutation (DRM) genotyping with both ViroSeq and American Type Culture Collection genotyping kits. DRM genotypes will be performed using leftover plasma collected for the routine blood tests at the time of the second failing VL on all patients with documented VF from both experimental and control arms of the trial.

All clinical data will be entered into paper-based forms. These paper forms will be transferred to data entry staff for inputting into program and project databases as relevant. Data quality checks will be made prior to data entry. The Data Manager will be responsible for uploading VL data into the networked computers for the pharmacists to access at the time of refill pick-ups to ensure that pharmacists are able to notify or remind patients that might require early repeat testing.

### Adverse event reporting

We do not anticipate significant adverse events related to the trial, as the Xpert HIV-1 VL test has been widely evaluated and never documented to pose more than minimal risk [11, 12, 15]. Instances of adverse events, either noted by clinicians or reported by the patients during scheduled or unscheduled clinical visits, will be documented by the clinicians. Any documented adverse events across the site, either for the existing HIV Care and Treatment program or for this trial, are collated by a designated pharmacist and reported monthly to the National Pharmacovigilance Centre, at the headquarters of the National Agency for Food and Drug Administration and Control in Abuja, Nigeria. Any potential study-related adverse events will also be reported to the PIs, the sponsor, and the Institutional Review Boards (IRBs) in accordance with the institutional and sponsor requirements.

### Outcome measures

The primary outcome measure for this trial is the proportion of patients with suppressed VL (< 1000 copies/mL) at month 12 post-initiation of ART in SOC VL versus POC VL. Secondary outcomes measures include comparisons between the two trial arms on: ART adherence patterns; loss to follow-up rates by 12 months post-initiation of ART; impact of trial site on virologic suppression rates; time from ART initiation to confirmation of VF; time from ART initiation to switch to 2 L treatment; time from specimen collection to availability of VL results in patient charts; time from specimen collection to delivery of VL results to patient; time from lab confirmation of first unsuppressed VL to adherence counseling provided and switch to 2 L; and, HIV DRM patterns in patients failing first line. We will also examine patient and HCW satisfaction with POC monitoring as part of our secondary analyses.

### Sample size estimation

Data from ongoing site databases were accessed to inform sample size calculations for the primary outcome. During 2004–2015, patient retention at 12 months post-initiation of ART for the site among HIV-infected patients that had no previous experience on ART ranged from 68 to 85%; VL suppression at month 12 post-initiation of ART ranged from 68 to 79%.

To detect an increase of at least 10% of patients with suppressed VL (from 70 to 80%) at month 12 following exposure to the POC arm, at a power of 80%, an alpha of 0.05 and using a two-tailed two sampled proportions Pearson’s Chi-squared test in Stata v.13.1, we estimated a minimal sample size of 294 was required per arm. To account for an average rate of 26% LTFU, acknowledging that rates might differ by group, we computed that we would need to enroll 794 patients (397 per arm) in order to adequately evaluate the difference in the primary outcome between trial arms.

### Statistical analysis

All quantitative statistical analyses will be conducted using Stata version 13.1 (StataCorp LLC, College Station, TX). For descriptive analyses, we will use standard univariate methods to display demographic variables (i.e., age, sex, education, occupation type, marital status), enrollment site, enrollment year, HIV transmission category, as well as clinical variables (i.e., hepatitis B virus and/or hepatitis C virus co-infection at enrollment, WHO clinical stage, ART regimen, CD4+ T-cell count, and VL). Basic bivariate analyses (Chi-square/Fisher’s exact for categorical variables; Wilcoxon or Kruskal-Wallis for continuous predictors, as relevant) will be conducted to determine if there are associations between predictor and outcome variables. Data collected on perceived facilitators and barriers to adherence and retention as potential predictors will be adapted for quantitative analyses to examine how the patients’ responses correlate with their outcomes. Based on the correlated variables in the first part of the descriptive analyses, variables will be collapsed by thematic areas to then create stratified variables that can be tested as predictors of the primary outcome. Multivariate modeling, to allow for controlling of confounders, will be conducted for any statistically significant associations identified in bivariate analyses.

LTFU at the 12-month time point will be defined as patients with no documentation of withdrawal, transfer, or death, but who have not made a monthly drug pick-up since their 9-month visit. Adherence will be estimated using electronic refill data, which has been shown to be a valid proxy [16–19]. We will compute average percent ART adherence as number of days supplied over total days in the given time interval, adjusting for amount of medication that should remain since last refill. Adherence will be evaluated as continuous variable and will also be collapsed into standard categories as previously published in the literature: ≥95%, 80–94.9, and < 80% [18, 20–22].

Differential LTFU rates across trial arms may compromise our ability to obtain a valid test of the null hypothesis of interest, if LTFU is associated with the endpoint within treatment arm. To address this concern, we plan to implement two supplemental analyses. First, we will implement an inverse-probability-of-censoring-weighted (IPCW) analysis, which will allow us to account for both baseline and post-randomization information on correlates of LTFU and the endpoint. We have implemented such an analysis in an observational study [23]. IPCW relies on the assumption that information available at baseline and at follow-up is sufficiently rich to account for dependent LTFU. Because we cannot guarantee such an assumption, in the event of a significant finding, we also plan to conduct a tipping point analysis, which aims to identify the potential response rate censored persons would have to experience to explain away the significant finding [24, 25]. This sensitivity analysis will be particularly insightful as it will directly inform on the robustness of a significant finding to potential bias due to dropout.

We will use the data collected in the HCW and patient post-trial surveys to evaluate the acceptability of POC versus SOC testing. Data will further be examined to evaluate associations with variables that might predict responses, including sex, age, site, occupation, marital status, and education level. The associations will be measured using standard bivariate analytic methods, including Chi-square or Fisher’s exact test for categorical variables and t-tests or Wilcoxon rank sum for continuous variables. Additionally, we will look at the correlations between adherence values and responses on whether or not POC VL monitoring encouraged patients to be more adherent to their regimen.

We do not anticipate a large percentage of patients in our trial to fail their ART regimens in the 12-month observation period. We conservatively estimate a 20% failure rate for all patients regardless of VL monitoring arm. DRM genotypes at the 12-month visit will be compared between POC and SOC VL monitoring arms. Because we only expect to have data on a small number of patients for this particular sub-analysis, the analyses will be largely descriptive in nature.

### Ethical considerations

All subjects will be explained about participation in the trial, including procedures, randomization, risks and benefits, and collection, use, and protection of personal data, and will need to provide written informed consent prior to enrollment. Written informed consent will be obtained for all patients that are enrolled in the trial. For children under 18 years of age, parental permission will be obtained from both parents if present at the clinic at time of consent, or one parent if only one is present. If both parents are deceased, unknown, or incompetent, consent will be obtained from the child’s legal guardian. Assent will be obtained from all children aged 7–17 years, which will also be documented in writing. As the trial duration for each patient is one year, if a patient who is 17 at the time of consent turns 18 during the course of the trial, they will be re-consented with the adult consent form. Written informed consent will also be obtained from HCWs participating in the post-trial survey. The trial protocol has been approved by the IRBs of JUTH and Harvard Chan.

### Data management and patient confidentiality

As part of the Harvard/APIN PEPFAR program, a relational electronic medical records system (EMRS) was developed in 2004 using the FileMaker Pro software [26]. The EMRS contains all data collected in the course of HIV care and treatment. Data are linked across database files through use of a unique ID code used for ART patients receiving care through the APIN PEPFAR program. The relational EMRS has a number of patient privacy and data security protections in place. As patients in the trial will continue in the SOC beyond the timeline of the respective evaluations, their routine clinical and laboratory data, including the VL test results, will be retained in their ongoing patient charts and electronic records indefinitely as part of their medical records. Electronic study data, including the VL test results obtained during the trial, relevant clinical data that are extracted from the patient’s medical records, and survey data will be de-identified and anonymized for the analyses.

Trial data will be managed by clinical trial staff at the sites under the supervision of the site PI. The PI will be responsible for ensuring the accuracy, completeness, legibility, and timeliness of the data reported. All personnel involved in this trial that will have access to patient data, as determined by the PIs, will be asked to complete a confidentiality agreement prior to the start of the trial enrollment and/or their participation in the overall project.

### Trial safety oversight

Clinical site monitoring is conducted to ensure that the rights and well-being of human subjects are protected, that the reported trial data are accurate, complete, and verifiable, and that the conduct of the trial complies with the currently approved protocol/amendment(s), with Good Clinical Practice guidelines, and with applicable regulatory requirements. The major monitoring and oversight of the trial safety will be managed by the relevant participating site IRBs and trial sponsor. No Data and Safety Monitoring Board has been established for this trial because the nature of this operational research study involves very minimal risk to the subjects, based on the definition under 45 CFR §46.102. The risk associated with participation in this trial is no greater than the risk the patient will be receiving in their SOC. Any modifications to the trial protocol will be communicated to all relevant parties, including the IRBs, trial participants, trial registry, and sponsors.

### Study staff training

In the weeks leading up to the study activation, job aids and protocol slides were prepared. Multiple training sessions were held with all levels of staff that would be participating in trial activities. These trainings were followed by walk-throughs of the trial activities with mock patients. Additionally, data staff tested all trial-specific databases to ensure they worked with mock data. Finally, the phlebotomist was trained on coordinating with laboratory staff for processing of POC samples that come in starting at the month 6 visit. Laboratory staff were trained on how to use the Xpert HIV-1 VL assay and completed their training by testing their skills using test strips provided by the U.S. Centers for Disease Control and Prevention (CDC).

### Quality assurance

The JUTH Laboratory Manager will oversee the quality of the POC testing for both JUTH and CHCZ sites. The oversight will include an electronic log of all instrument malfunctions, indeterminate results, and other performance issues. For monitoring quality control (QC) for the SOC Roche procedures, the CDC provides VL proficiency testing and external quality assessments (EQA) twice a year through Afriqual. CDC EQA will continue through the duration of this trial.

## Discussion

While the international community has long-awaited the development of POC VL assays, their availability is only the first step. As we have learned from the experience with POC HIV diagnostics, the successful use of these innovations requires careful consideration of acceptability, training, cost-efficiency, sustainability and QC; the mere availability of the POC test does not automatically ensure its adoption or scale-up; it must be successfully deployed in the clinical setting to truly achieve the “POC” benefit [27]. Therefore, the development of a “program for POC VL monitoring” can be informed by the proposed clinical trial in an appropriate LMIC ART setting. The results of this trial may demonstrate the value and feasibility of POC VL monitoring for broader implementation to support optimizing laboratory-clinic networks and patient care, which may facilitate monitoring of the third 90–90-90 target towards an AIDS-free world.

### Strengths

In addition to addressing questions about the efficacy of POC versus SOC VL monitoring, we believe that the way this trial has been designed will allow the investigators to address a number of important issues related to implementation. The study design will allow for us to not only answer questions regarding the feasibility and acceptability, but it will also provide empirical data on TAT and impact on time to making clinical decisions that can potentially impact patient outcomes. Furthermore, for patients that are failing ART, we will conduct a descriptive evaluation of impact of TAT on development of DRMs. This study also allows for a comparison of implementation impact at a tertiary versus secondary treatment site.

More specifically, the primary outcome as designed will allow for a direct measure of use of the test on patient outcomes. The majority of studies examining POC assays are focused on comparing results from the new assay to a gold standard or the existing standard-of-care method of measurement. In this trial, we have focused our primary outcome on examining if implementation of POC VL testing will also translate into higher percentage of patients with a suppressed VL. As such, not only will this study design address the question if this assay can be used as a means to increase access to VL testing in RLS, but also if the use of POC VL testing make a contribution in getting closer to the third 90–90-90 goal of 90% suppression.

The secondary outcomes in this trial will address questions about the POC technology itself as we will gather data on the robustness of the equipment, number of invalid tests, and number of equipment malfunctions. A major concern with POC technologies is maintenance of high quality and, therefore, a successful POC program must develop sustainable policies for QC [28]. Second, we can use this trial to examine potential issues related to staff training and operator performance. While these assays have been designed to require minimal technical training, the actual operation over time may reveal performance problems that were not identified in the developer’s laboratory. A third, but important, issue that we also can address is patient acceptability. A relatively recent study examining acceptability of other POC HIV rapid tests indicates that many felt that the POC assay was less accurate than tests conducted in the standard laboratory [27]. Therefore, by directly querying patients in the POC arm about their thoughts on the accuracy of the results, we will have better information regarding patient acceptability of POC VL assays that will be useful in understanding patient uptake and ultimate outcomes. Finally, this trial will also allow for us to examine impact on patient outcomes with concomitant measurement of impact on patient and clinician time, which are important indicators of how a new test will ultimately be incorporated into practice.

### Potential limitations

There are some potential limitations associated with this trial. As is the nature of patients enrolled in a clinical trial, they might be more inclined to be compliant to treatment protocols than those not being so closely monitored; as such, it is possible that the adherence and retention values for patients in both groups will be higher than they might be if these patients were not enrolled in a trial. A second major limitation is that we have based our calculations on prior year enrollment numbers in order to estimate when we will meet our target enrollment. If enrollment rates change over time, it is possible that we will not meet our enrollment goals.

### Potential implications for practice

The proposed clinical trial will demonstrate if POC VL monitoring is feasible and acceptable in a typical Nigerian ART center setting. Analysis of results of the 12-month trial will indicate if POC VL monitoring can improve viral suppression rates after 12 months on ART. It is also possible that our trial will indicate whether POC versus SOC VL monitoring will improve patient retention on ART and promote better ART adherence. If results on its efficacy are promising, POC VL monitoring could then be integrated into Nigerian ART policy for more widespread implementation in HIV care and treatment programs across the country.

### Trial status

Patient recruitment started on April 9, 2018 and the anticipated date of enrollment closure is October 9, 2019.

## Abbreviations

2 L: second-line
AIDS: Acquired immunodeficiency syndrome
APIN PHI: AIDS Prevention Initiative Nigeria - Public Health Initiatives
ART: Antiretroviral treatment
CDC: Centers for Disease Control and Prevention
CHCZ: Comprehensive Health Centre, Zamko
DRM: Drug resistance mutation
EMRS: Electronic medical records system
EQA: External quality assessment
FWA: Federal Wide Assurance
HCW: Health care worker
HIV: Human immunodeficiency virus
ID: Identification
IPCW: Inverse-probability-of-censoring-weighted
IRB: Institutional review board
JUTH: Jos University Teaching Hospital
LMIC: Low- and middle-income countries
LTFU: Loss to follow-up
PEPFAR: President’s Emergency Plan for AIDS Relief
PI: Principal Investigator
POC: Point-of-care
QC: Quality control
RLS: Resource-limited settings
Roche VL: Roche AmpliPrep/COBAS TaqM (CAP/CTM) HIV-1 test, v2.0
SOC: Standard of care
TAT: Turnaround time
TB: Tuberculosis
UNAIDS: Joint United Nations Programme on HIV/AIDS
VF: Virologic failure
VL: Viral load
WHO: World Health Organization
Xpert HIV-1 VL: Cepheid Xpert HIV-1 Viral Load

## References

[1] Joint United National Programme on HIV/AIDS (2015). Ambitious treatment targets: writing the final chapter of the AIDS epidemic.

[2] World Health Organization. HIV diagnostic tests in low- and middle-income countries: forecasts of global demand for 2014–2018. Geneva; 2015. Available from: http://apps.who.int/iris/bitstream/10665/179864/1/9789241509169_eng.pdf?ua=1

[3] World Health Organization. The availability and use of HIV diagnostics: a 2012/2013 WHO survey in Low- and middle-income countries. Geneva; 2014. Available from: http://apps.who.int/iris/bitstream/10665/147213/1/9789241507905_eng.pdf

[4] UNAIDS. Country factsheets: Nigeria, 2016. 2016. Available from: http://www.unaids.org/en/regionscountries/countries/nigeria.

[5] Federal Ministry of Health National AIDS/STIs Control Porgramme (2014). Integrated national guidelines for HIV prevention treatment and care. Abuja.

[6] Lofgren SM, Morrissey AB, Chevallier CC (2009). Evaluation of a dried blood spot HIV-1 RNA program for early infant diagnosis and viral load monitoring at rural and remote healthcare facilities. AIDS..

[7] Saito S, Duong YT, Metz M (2017). Returning HIV-1 viral load results to participant-selected health facilities in national population-based HIV impact assessment (PHIA) household surveys in three sub-Saharan African countries, 2015 to 2016. J Int AIDS Soc.

[8] Lecher S, Ellenberger D, Kim AA (2015). Scale-up of HIV viral load monitoring--seven sub-Saharan African countries. MMWR Morb Mortal Wkly Rep.

[9] Lecher S, Williams J, Fonjungo PN (2016). Progress with scale-up of HIV viral load monitoring - seven sub-Saharan African countries, January 2015-June 2016. MMWR Morb Mortal Wkly Rep.

[10] Ceffa S, Luhanga R, Andreotti M (2016). Comparison of the Cepheid GeneXpert and Abbott M2000 HIV-1 real time molecular assays for monitoring HIV-1 viral load and detecting HIV-1 infection. J Virol Methods.

[11] Gous N, Scott L, Berrie L, Stevens W (2016). Options to expand HIV viral load testing in South Africa: evaluation of the GeneXpert(R) HIV-1 viral load assay. PLoS One.

[12] Moyo S, Mohammed T, Wirth KE (2016). Point-of-care Cepheid Xpert HIV-1 viral load test in rural African communities is feasible and reliable. J Clin Microbiol.

[13] World Health Organization. WHO prequalification of in vitro diagnostics, pubilc report, product: Xpert HIV-1 viral load wtih GeneXpert, version 2.0. Geneva; 2017. Available from: http://www.who.int/diagnostics_laboratory/evaluations/pq-list/hiv-vrl/170720_final_pq_report_pqdx_0192_0193_0194_0195_070-00.pdf?ua=1

[14] Federal Ministry of Health - National AIDS and STIs Control Programme (2016). National guidelines for HIV prevention, treatment and care.

[15] Nash M, Huddart S, Badar S, et al. Performance of the Xpert HIV-1 viral load assay: a systematic review and meta-analysis. J Clin Microbiol. 2018;56. 10.1128/JCM.01673-17 PMC5869835 29386266

[16] El-Khatib Z, Katzenstein D, Marrone G (2011). Adherence to drug-refill is a useful early warning indicator of virologic and immunologic failure among HIV patients on first-line ART in South Africa. PLoS One.

[17] Grossberg R, Gross R (2007). Use of pharmacy refill data as a measure of antiretroviral adherence. Curr HIV/AIDS Rep.

[18] Nachega JB, Hislop M, Dowdy DW (2006). Adherence to highly active antiretroviral therapy assessed by pharmacy claims predicts survival in HIV-infected south African adults. J Acquir Immune Defic Syndr.

[19] Sangeda RZ, Mosha F, Prosperi M (2014). Pharmacy refill adherence outperforms self-reported methods in predicting HIV therapy outcome in resource-limited settings. BMC Public Health.

[20] Goldman JD, Cantrell RA, Mulenga LB (2008). Simple adherence assessments to predict virologic failure among HIV-infected adults with discordant immunologic and clinical responses to antiretroviral therapy. AIDS Res Hum Retrovir.

[21] Low-Beer S, Yip B, O'Shaughnessy MV, Hogg RS, Montaner JS (2000). Adherence to triple therapy and viral load response. J Acquir Immune Defic Syndr.

[22] Weidle PJ, Wamai N, Solberg P (2006). Adherence to antiretroviral therapy in a home-based AIDS care programme in rural Uganda. Lancet..

[23] Scarsi KK, Eisen G, Darin KM (2016). Superior effectiveness of zidovudine compared with tenofovir when combined with nevirapine-based antiretroviral therapy in a large Nigerian cohort. Clin Infect Dis.

[24] Molenberghs G, Kenward M (2007). Missing data in clinical studies.

[25] Panel on Handling Missing Data in Clinical Trials; National Research Council. The prevention and treatment of missing data in clincial trials. Washington, D.C.; 2010.

[26] Chaplin B, Meloni S, Eisen G (2015). Scale-up of networked HIV treatment in Nigeria: creation of an integrated electronic medical records system. Int J Med Inform.

[27] Pai NP, Vadnais C, Denkinger C, Engel N, Pai M (2012). Point-of-care testing for infectious diseases: diversity, complexity, and barriers in low- and middle-income countries. PLoS Med.

[28] Fonjungo PN, Osmanov S, Kuritsky J (2016). Ensuring quality: a key consideration in scaling-up HIV-related point-of-care testing programs. AIDS..

[29] National Health Research Ethics Committee of Nigeria. (NHREC). National Code of health. Research Ethics. 2007; Available from: http://www.nhrec.net/nhrec/NCHRE_10.pdf.

